# The enhancing effects of alcoholic extract of Nigella sativa seed on fertility potential, plasma gonadotropins and testosterone in male rats

**Published:** 2012-07

**Authors:** Rahmatollah Parandin, Namdar Yousofvand, Rostam Ghorbani

**Affiliations:** 1 *Department of Animal Physiology-Biology, Faculty of Sciences, Payame Noor University of Tehran, Tehran, Iran.*; 2 *Department of Biology, Faculty of Sciences, Razi University of Kermanshah, Kermanshah, Iran.*; 3 *Center of Fertility and Infertility, Kermanshah University of Medical Sciences, Kermanshah, Iran.*

**Keywords:** *Nigella sativa*, *Fertility*, *Male rat*

## Abstract

**Background:** The task force on plants for fertility regulation in men continued with its program to identify novel prototypes in plants alleged to have fertility regulating properties. *Nigella Sativa* seeds are frequently used in folk medicine in the Middle East and some Asian countries for the promotion of good health and treatment of many ailments.

**Objective:** To evaluated the role of alcoholic extract of *Nigella sativa* on fertility potential, Pituitary-testicular axis hormones and Testosterone in male rats.

**Materials and Methods:** 24 male rats were randomly divided into 3 groups; control, group A and group B, each group comprising of 8 rats. Animals in control group received 1 ml of normal saline and treatment groups (A and B) received (gavage) graded doses of 200 and 400 mg/kg body weight of alcoholic extract of* Nigella sativa* seeds on a daily basis for 60 days. At the end of treatment period, fertility parameters such as body and reproductive organs weight, sperm motility, viability and count, epididymal sperm reserve (ESR), daily sperm production (DSP), blood testosterone concentration, Gonadotropins levels and fertility index were measured.

**Results:** There was a significant difference in testes and epididymidis weight, sperm count, ESR, DSP, blood testosterone concentration, LH and fertility index in both the lower dose group and the higher group as compared to the control group.

**Conclusion:** The results of this study showed that alcoholic extract of *Nigella sativa* seed especially in higher doses could increase fertility potential, LH and testosterone concentration in male rats.

## Introduction

Infertility is a complex disorder with significant medical, psychosocial and economic aspects (1). About 25% of couples do not achieve pregnancy within 1 year, 15% of whom seek medical treatment for infertility and less than 5% remain unwillingly childless. Infertility affects both men and women. Male causes for infertility are found in 50% of involuntarily childless couples (2).

A wide majority of medicine plants possess pharmacological principles, which has rendered them useful as curatives for numerous ailments. According to the World Health Organization (WHO) reports, 70-80% of the world population confide in traditional medicine for primary health care (3). Plants and derivatives of plant played a key role in world health and have long been known to possess biological activity. Thirty percent of all modern drugs are derived from plants (4). In addition, Plants have a long folklore of use in aiding fertility, including fertility-enhancing properties and aphrodisiacal qualities (5-6). 

Nigella sativa L. belong to the botanical family of ranunculaceae (7). It has been known as black seed and its seeds are frequently used in folk medicine in Middle East and some Asian countries for the promotion of good health and treatment of many ailments (8-9). 

N. sativa (Cyah-daneh in Persian) seeds has been used in traditional Iranian medicine as a natural remedy for promotes females menstruation, galactagogue, carminative, laxative, etc. and anti-parasitic properties (10-11). Recently, animal studies have been shown that extracts of N. sativa seeds have many therapeutic effects such as gastroprotective (12), Anti-tumor (13, 14), Anti-anxiety (15), Anti-microbial (16, 17), Anti-inflammatory (18) and Anti-Oxidant (19-22).

The seed of N. sativa has many different chemical components, including mucilage, crude fiber, Reducing sugars, resins, alkaloids, flavnoids, organic acids, sterols, Tannins, Saponins, and proteins (23-24). In addition, it has a high content of unsaturated fatty acids, especially Linoleic acid (55.6%), Oleic acid (23.4%) and Palmitic acid (12.5%) (25). It is known that the biological activity of N. sativa seeds is attributed to its essential oil components (26). 

The main compounds contained are Thymoquinone (30-48%), P-cymene (7-15%), Carvacrol (6-12%), 4-terpineol (2-7%), T-anethole (1-4%) and Sesquiterpene (1-8%) (27). Thymoquinone and its derivatives are the most putative pharmacologically active constituents of N. sativa (28). However, the effects of N. sativa seeds on fertility parameters are not enough. Samir Bashandy (29) shown that administration of N. sativa oil to hyperlipidemic rats improved their reproductive efficiency and produced additional protection against hyperlipidemia induced reduction in fertility. Also, Mukhallad *et al* (30) concluded that the aqueous extracts of Nigella sativa have increased spermatogenesis of male albino rats. 

In addition, El-Tahomi *et al* (31) shown that the inclusion of a mixture of equal quantities from radish, rocket and black cumin (N. sativa) meals on the expense of approximately 50% soybean meal protein improved the semen characteristics and reduced free radicals in the seminal plasma. Therefore, regardless to value of plant used in traditional medicine for drug discovery of fertility-enhancing, this study was conducted to examine the effect of alcoholic extract of Nigella sativa seed on fertility potential, plasma Gonadotropins and testosterone in male rats. 

The advantages of this study has been in compared to the previous studies, using seed alcoholic extract and parameters such as DSP, ESR and Gonadotropins level that these parameters were not used in previous studies(29-31). 

## Materials and methods

**Plant materials and extraction procedure**

The seeds of *N. sativa* were purchased from a local herb market and the taxonomic identification of the seeds was confirmed by a senior plant taxonomist. The extract was prepared according to WHO protocol CG-04 (32). For the preparation of an alcoholic extract, the seeds were dried, powdered and then subjected to soxhelet apparatus for extraction with 50% ethanol. The extract obtained was filtered and then evaporated to dryness under reduced pressure which yielded about 8.5% of solid residue.

**Animals**

In this experimental study, 24 adult male Wistar rats 3 months old weighting between 230-270 gm were bred in the animal house at Payame-Noor university of Kermanshah province. Animals were maintained under controlled temperature of 23±2^o^C and 12 hr light/12 hr darkness in plastic cages. Food and water were available ad labitum.

**Treatment**

Male animals of proven fertility were divided randomly into three groups: Control group received vehicle (normal saline) for 60 days and treatment groups A and B, received the extract of *N. sativa* 200 and 400 mg/kg body weight for 60 days (30) by orally (gavage). 

**Experimental design**

Body weight: The body weight changes of each group were defined as the difference between first and last days of treatment.

**Fertility index (mating test)**

After 24 hr of the last dose, all male rats caged separately with two coeval untreated females of proven fertility of the same strain for 10 days during which two estrus cycles should have elapsed. Ten days later, the mated females killed by cervical dislocation under light ether anesthesia. During autopsy, uterus and both the uterine horns were examined for the number of implantation sites and the ovaries were excised and examined for the number of fresh corpora lutea using a stereomicroscope (Olympus, Japan). Fertility index was expressed as the ratio of the number of implantation sites to the number of corpora lutea (33).

Organs weight: Testes and epididymis removed and weighted with digital balance (Sartorius, Germany, 0.001 gram readability).

Sperm motility and count: To determine sperm motility and sperm counts, 100 mg of caudal epididymis was minced in 5 ml of physiological saline. One drop of an evenly mixed sample was applied to a Neubauer’s counting chamber under a cover slip. Quantitative motility expressed as a index was determined by counting both motile and immotile spermatozoa per unit area. Epididymal counts was made by routine procedure and expressed as million/ml of suspension (34). 

Sperm viability: To determine sperm vitality, 40 μl of freshly liquefied semen was thoroughly mixed with 10 μl of eosin-nigrosin (Merck, Germany), and 1 drop of this mixture was transferred to a clean slide. At least 200 sperms were counted at a magnification of ×100 (Olympus Japan) under oil immersion. Sperms that were stained pink or red were considered dead, and those unstained were considered viable (35-36).

Daily sperm production (DSP) and Epididymal sperm reserve (ESR): To determine DSP, after the tunica albuginea had been removed from one testis, Parenchyma was homogenized in 20 ml of 0.9% saline-0.05% (v/v) Triton X-100 solution (STS) for 1 minute by a homogenizer. After a dilution of 1/10, Counts for 8 hemocytometer chambers were averaged. The DSP and its efficiency (DSP per gram of testis) were determined by division of the elongated spermatid count per testis and spermatids per gram of testis by 6.3, the duration of steps 17-19 spermatids in the seminiferous epithelial cycle for rats. The epididymal sperm transit rate was calculated by dividing the cauda epididymal sperm number by the DSP (37-39). To determine ESR, one epididymis was minced and homogenized in 50 ml STS fluid for 2 min. The number of spermatozoa in each homogenate was determined as DSP (37-38).

Hormone assay: At the end of experiments, blood was collected by cardiac puncture. Serum was separated by centrifugation at 3000 rpm for 15 min and stored frozen at -20^o^C until use. Plasma follicle-stimulating hormone (FSH), luteinizing hormone (LH) and testosterone were measured by radioimmunoassay (RIA) using special kits (Radim, Italy) as described in the instructions provided with the kits. 

**Statistical analysis**

Values were presented as mean±SEM using statistical software of GraphPad Instat V2.01. Statistical analysis was carried out by one-way analysis of variance (ANOVA) and the comparison between the control and experimental groups was done using the Tukey-Kramer test. 

## Results

Body weight and weight of reproductive organs: There was no a significant difference in body weight in control group and treated rats with *N. sativa*, but at the higher dose level (400 mg/kg body weight) resulted in significant increase in the testes (p<0.05) and epididymis (p<0.01) weights (Table I).

Sperm motility, viability and count:* N. sativa* extract treatment had no effect on the sperm motility and viability of the rats observed. Extract administration caused a significant increase in sperm count of lower dose (p<0.05) group and higher dose (p<0.001) group (Table II). 

ESR and DSP: Administration of 400 mg/kg body weight extract showed a significant increase (p<0.01) in ESR of treated group as compare to the control. There was a significant increase in DSP both in 200 (p<0.05) and 400 (p<0.01) mg/kg body weights treated animals as compare to the control (Table II).

Hormone levels assay: Plasma levels of testosterone and LH were significantly increased in treatment groups compared to the control. In regard to, testosterone concentration, the changes were more prominent in the higher dose. There was no significant difference in FSH levels of treated rats as compared with the control (Table III).

Fertility index: There was a significant increase in fertility index in treatment groups (p<0.05 and p<0.01) compared to the control especially in higher dose (Table III).

**Table I:** Effects of alcoholic extract of *Nigella sativa* seeds on body and reproductive organs weight (n=8).

**Group**	**Body weight (gm)**	**Testes (mg/kg body weight)**	**Epididymis (mg/kg body weight)**
Control	61.37 ± 3.57	638.62 ± 19.72	244.75 ± 14.74
200 mg/kg	58.62 ± 4.28	664.25 ± 25.11	253.37 ± 12.36
400 mg/kg	64.12 ± 3.2	735.5 ± 32.79*	331.12 ± 1948**

*: p<0.05,

** : p<0.01, compared with the control.

**Table II:** Effects of alcoholic extract of *Nigella sativa* seeds on sperm indexes (n=8).

**Group**	**Motility (%)**	**Viability (%)**	**Sperm count (million/ml)**	**)** **ESR)** 1 **(million)**	**)** **DSP)** 2 **(million)**
Control	77.5 ± 3.44	80.37 ± 3.56	54.87 ± 1.9	245.5 ± 12.6	14.62 ± 0.88
200mg/kg	72.25 ±3.52	76.62 ± 2.69	61.87 ± 1.84*	249.87 ±10.84	18.37 ± 0.75*
400mg/kg	81.75 ± 2.41	84.5 ± 2.93	66.25 ± 1.6***	300.12 ± 8.49**	19.25 ± 0.84**

* : p<0.05,

** : p<0.01,

*** : p<0.001, compared with the control.

1 - DSP: Daily sperm production,

2 - ESR: Epididymal sperm reserve.

**Table III:** Effects of alcoholic extract of *Nigella sativa* seeds on hormones and fertility index (n=8).

**Group**	**Testosterone ** **)** **ng/ml)**	**FSH (mIU/ml)**	**LH (mIU/ml)**	**Fertility index (%)**
Control	2.62 ± 0.12	2.12 ± 0.11	0.845 ± 0.02	69.87 ± 2.44
200mg/kg	2.76 ± 0.11*	2.16 ± 0.15	0.991 ± 0.04*	80.87 ± 3.11*
400mg/kg	3.21 ± 0.17***	2.31 ± 0.11	0.961 ± 0.03*	85.25 ± 2.72**

* : p<0.05,

** : p<0.01,

*** : p<0.001, compared with the control.

## Discussion

This research demonstrates that oral administration of alcoholic extract of *N. sativa* doses 200 and 400 mg/kg body weight in male rats for 60 days caused a significant increase in some fertility parameters especially in higher dose. The model employed in this work has been used previously by several investigators to assess the effects of different compounds on fertility and reproduction in laboratory animals (39-41). 

Treatment of rats with *N. sativa* seed alcoholic extract did not affect in body weight. Also, regardless to the side effects of *N. sativa*, previous studies have been shown that there were no toxic effects or inflammatory reaction (18, 42-43). This suggests that this extract at the applied doses has no general toxic effect on body weight. The present study showed that testes and epididymis weight at dose 400 mg/kg significantly increased and this effect was seen in previous studies (29-31). 

The testes, epididymis and other reproductive organs are structurally and physiologically dependent upon the testosterone and other androgens. Testosterone stimulates growth and secretary activity of the reproductive organs (44-46) so a significant increase of these hormones in our study could increase the number and function of somatic and germinal cells of testis and in results increase the testis and epididymis weight. 

The extract significantly increased sperm count and DSP especially in higher dose without significant changes in the sperm motility and viability. In previous study, ESR and DSP have been not assayed but sperm count, motility and viability had a significant increase (29-31). It is a well confirmed that, these parameters in mammals are regulated by the two Gonadotropins, LH and FSH. FSH binds with receptors in the sertoli cells and directly stimulates spermatogenesis. 

LH stimulates the production of testosterone in Leydig cells, which in turn may act on the Sertoli and peritubular cells of the seminiferous tubules and indirectly stimulates spermatogenesis via testosterone (44-46). Therefore, a significant increase in LH hormone concentration in our study treated rats could lead to increased testosterone secretion from Leydig cells (46). 

At previous study shown that N. sativa (300 mg/kg body weight for 60 day) increased the number of Leydig cells and its diameter nuclear in rat testes (30). However, this study showed that the FSH levels remained unaltered. It is possible that testis seminiferous tubules induced directly by the N. sativa extract or indirectly by testosterone and stimulated sperm counts and DSP. Increase in fertility index of untreated female’s rats which were mated with treated males could be due to of fertility parameters enhancement as indicated by the sperm count, ESR and DSP. 

The present study showed that ESR at dose 400 mg/kg significantly increased. The epididymis, a natural sperm reservoir, has maturational and storage functions and it protects spermatozoa from oxidative injury by encouraging scavengers of reactive oxygen species (47). It has been observed that rats treated for 8 weeks with ascorbic acid, a potent antioxidant, showed a significantly increased epididymal sperm concentration (48). Treatment with isoflavones resulted in an increase in sperm count and antioxidant activity in male rabbit (49). 

Moreover, when piperine (an alkaloid present in the fruits of Black Pepper) or Cannabinoids (one of the oldest narcotic drugs of plant origin), were administered to rats, there were a decrease in the activity of antioxidant enzymes in the epididymis and reduced spermatogenesis and epididymal sperm count (50-51). Consistent with this notion, several investigators had demonstrated that Nigella sativa has exceptionally high antioxidant properties (19-22). 

In addition, these observations may be connected with other chemical composition of this plant. Phytochemical analysis indicated the rich presence of unsaturated fatty acids (Linoleic acid 55.6%, Oleic acid 23.4%, Palmitic acid 12.5%, Stearic acid 3.4% and else.) in N. sativa seeds (25). Fellner *et al* shown that supplementation of rats diets with oils rich in polyunsaturated fatty acids, such as Linoleic acid has positively influenced reproductive functions (52). 

Also, Gromadzka-Ostrowska *et al* shown that the unsaturated fatty acids stimulate the activity of 17 β-hydroxysteroid dehydrogenase, the most important key enzyme in the testosterone biosynthesis pathway (53). Moreover, Samir Bashandy found that administration of N. Sativa oil to hyperlipidemic rats improved their reproductive efficiency and produced additional protection against hyperlipidemia induced reduction in fertility (29). 

In addition, Thymoquinone is the major active component derived from Nigella sativa and many of the pharmacodynamic effects reported above for N. sativa are due to Thymoquinone (54). Gokce *et al* (55) has been confirmed that Thymoquinone treatment has protective effects on testicular parameters. 

## Conclusion

From the observation in this study, we conclude that the alcoholic extract of *Nigella sativa *seeds (200 and 400mg/kg body weight) for 60 days caused a positive effect on some fertility parameters and pituitary-testicular hormone axis in especially higher dose. These effects may be connected with the chemical composition of plant. 

However, it appears that the primary site of *N. Sativa* action may be on the brain or gonads and further studies are required to clarify these points.
